# Patient characteristics in sepsis-related deaths: prevalence of advanced frailty, comorbidity, and age in a Norwegian hospital trust

**DOI:** 10.1007/s15010-023-02013-y

**Published:** 2023-03-09

**Authors:** Marianne Ask Torvik, Stig Haugset Nymo, Ståle Haugset Nymo, Lars Petter Bjørnsen, Hanne Winge Kvarenes, Eirik Hugaas Ofstad

**Affiliations:** 1 Department of Infectious Disease, Nordland Hospital Trust, Parkveien 95, 8005 Bodø, Norway; 2 Department of Emergency Medicine, Nordland Hospital Trust, Parkveien 95, 8005 Bodø, Norway; 3 Department of Cardiology, Nordland Hospital Trust, Parkveien 95, 8005 Bodø, Norway; 4 Emergency Department, Clinic for Emergency Medicine and Prehospital Care, St. Olav’s Hospital University Hospital, NTNU, ISB, Prinsesse Kristinas gate 3, 7030 Trondheim, Norway; 5 UiT the Arctic University of Norway, Faculty of Health Sciences, Hansine Hanses vei 14, 9037 Tromsø, Norway

**Keywords:** Sepsis, Mortality, Frailty, Comorbidity

## Abstract

**Objective:**

To examine the prevalence of advanced frailty, comorbidity, and age among sepsis-related deaths in an adult hospital population.

**Methods:**

Retrospective chart reviews of deceased adults within a Norwegian hospital trust, with a diagnosis of infection, over 2 years (2018–2019). The likelihood of sepsis-related death was evaluated by clinicians as sepsis-related, possibly sepsis-related, or not sepsis-related.

**Results:**

Of 633 hospital deaths, 179 (28%) were sepsis-related, and 136 (21%) were possibly sepsis-related. Among these 315 patients whose deaths were sepsis-related or possibly sepsis-related, close to three in four patients (73%) were either 85 years or older, living with severe frailty (Clinical Frailty Scale, CFS, score of 7 or more), or an end-stage condition prior to the admission. Among the remaining 27%, 15% were either 80–84 years old, living with frailty corresponding to a CFS score of 6, or severe comorbidity, defined as 5 points or more on the Charlson Comorbidity Index (CCI). The last 12% constituted the presumably healthiest cluster, but in this group as well, the majority died with limitations of care due to their premorbid functional status and/ or comorbidity. Findings remained stable if the population was limited to sepsis-related deaths on clinicians’ reviews or those fulfilling the Sepsis-3 criteria.

**Conclusions:**

Advanced frailty, comorbidity, and age were predominant in hospital fatalities where infection contributed to death, with or without sepsis. This is of importance when considering sepsis-related mortality in similar populations, the applicability of study results to everyday clinical work, and future study designs.

**Supplementary Information:**

The online version contains supplementary material available at 10.1007/s15010-023-02013-y.

## Introduction

Sepsis is a common cause of health loss and death worldwide [1, 2]. In the United States, sepsis has been found to contribute to every two-to-three hospital deaths [3]. In Norway, a high-income country (HIC) with long life expectancy [4], the most recent national data estimated that sepsis accounted for 13% of hospital deaths, with a hospital mortality for sepsis admissions of 19% [5].

To improve patient outcomes, extensive research, awareness campaigns, and improvement programs for sepsis recognition and treatment have been published and implemented, especially over the last decade. However, the extent to which sepsis mortality among adults of HICs has changed has been debated, as randomized controlled trials (RCTs) have not found significant improvement [6, 7]. Considering this, some recent studies have addressed the preventability of many sepsis-related deaths, and the attributable fraction of mortality from sepsis in sepsis-related deaths [8–10].

Rhee et al*.* had experienced clinicians estimate the preventability of sepsis-related deaths in 300 patients within 6 US hospitals in 2014 and 2015 [8]. They found that 12% of sepsis-related deaths were possibly or definitely preventable if the patient had received optimal in-hospital care. However, in an invited commentary to Rhee et al.’s article, Laura Evans points out that in their study, the included hospitals had much higher compliance to guideline care for sepsis than most other hospitals in the US, potentially underestimating the preventability of sepsis death in other hospitals. She also points out the low interrater reliability in judging preventability in the study, encouraging caution in interpreting their findings [11].

Shankar-Hari et al. estimated the attributable fraction of mortality from sepsis to inform future sepsis study designs, which they found to be 15% in a large, propensity-matched intensive-care unit (ICU) population in the UK [10]. Kopczynska et al. estimated the fraction of patients in Welsh ward populations in 2016 and 2017, who fulfilled sepsis criteria and died within 3 months, which on clinical review were considered to die from sepsis [9]. They found that overall, 24% of deaths were attributable to sepsis. Among these, 78% were living with frailty corresponding to a Clinical Frailty Scale (CFS) score of 6 or more, and 70% had existing do not attempt cardiopulmonary resuscitation (DNA-CPR) orders.

Singer et al. argued in a commentary to the Lancet in 2019, partly based on these studies, that the high prevalence of comorbidity and frailty make most sepsis-related deaths neither attributable to sepsis nor preventable [12]. However, their argument has been met by critics pointing out that there are several register, prospective, and observational studies demonstrating improvement in mortality rates with improvement in care [13, 14] as well as questioning if the data underlying Singer et al.’s argument are representative and sufficient to support their claims [15].

Our study aims to contribute to this debate by providing a thorough descriptive analysis of patient characteristics in sepsis-related hospital deaths among adults in our region. We believe that these investigations are of importance when considering sepsis-related mortality in similar populations, the applicability of study results to everyday clinical work, and future study designs.

As previous studies have demonstrated that many patients who died from sepsis were living with advanced frailty, comorbidity, and age, our study tries to emphasize how many—and who—were not.

## Materials and methods

### Study design and setting

Retrospective chart reviews in a Northern Norwegian hospital trust, comprising two local and one regional hospital. The three hospitals serve a population of 160 000 inhabitants as local hospitals and 240 000 inhabitants as regional center for, e.g., intensive care, invasive cardiology, and infectious diseases. Transfers to university hospitals from our hospital’s ICU were included if transferred due to infection. The study period followed a period of quality improvement measures focusing on early administration of antibiotics if sepsis was suspected.

### Selection of participants

**Fig. 1:**
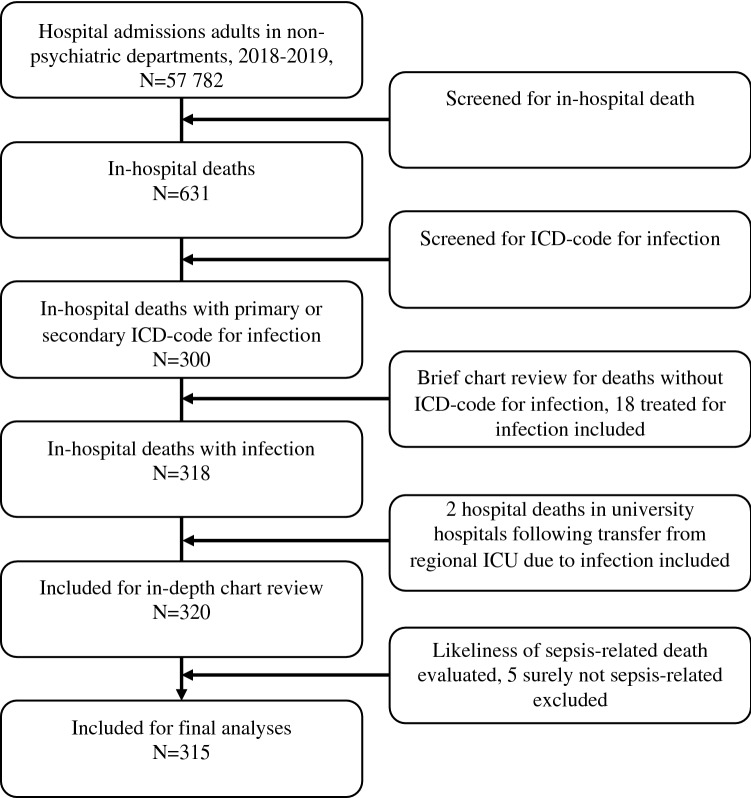
Selection of participants

The selection process is presented in Fig. 1. *Study population*: All adults ≥ 18 years of age in non-psychiatric departments who died in-hospital during 2018–2019 with a primary or secondary diagnosis of infection. There were 631 deaths among 57,782 hospital admissions, and 93% of these deaths occurred during emergency admissions. For each year, the number of admissions was about twice the number of unique patients (28,821 and 13,613 in 2018, 28,961 and 13,664 in 2019, respectively). Cases were first identified by hospital administrative staff as discharged as deceased, and then screened for a primary or secondary diagnosis of infection, applying a list of 283 International Statistical Classification of Diseases (ICD-10) codes for infection, based on previously published lists for similar use [5, 16]. Brief chart reviews were then performed for hospital deaths without ICD-10 codes for infection, including those treated for infection, but not coded accordingly. Transfers from the regional hospital’s ICU to university hospitals were collected from the ICU’s local database, charts were reviewed, and cases were included if transferred due to an infection-related condition (e.g., need for extracorporeal membrane oxygenation, ECMO) and deceased in a university hospital. Ultimately, the likelihood of sepsis-related death was evaluated by clinicians, and cases were excluded if they were without doubt not sepsis-related deaths, as defined in *Measurements*. *Control populations:* Two control populations were defined. (1) Sepsis survivors: A randomized draw of adults alive > 30 days after discharge with a primary or secondary diagnosis of infection, who fulfilled the Sepsis-3 criteria during their hospital admission, defined as a rise in the Sequential Organ Failure Assessment (SOFA)-score by two or more points during the worst 24-h period. Hospital administrative staff identified 10 419 ICD-10 codes for infection for patients discharged alive during the study period. From these, 500 random cases were drawn, of which 342 charts were reviewed to identify 50 includable cases. The same individual could hypothetically be eligible for both the study and the control population: If they were hospitalized with sepsis and discharged alive, but then > 30 days later hospitalized with sepsis again and deceased, they were eligible for the randomized draw to the control group through the first hospitalization and included in all hospital deaths through the last hospitalization. This did not occur. (2) Deceased patients without infection: A randomized draw of 50 out of the 316 adults deceased in-hospital during the study period, who were not diagnosed with an infectious disease during the admission.

### Measurements

*Raters and raters’ agreement:* Chart reviews were performed primarily by the authors, who are residents and consultants in internal and emergency medicine. Medical students coded nonevaluative data after a training period with parallel coding alongside the authors. Raters’ agreement for evaluative data was calculated with Cronbach’s alpha after the initial parallel coding by four authors, resulting in 0.93 for CFS and 0.90 for evaluation of sepsis-related death (excellent ≥ 0.90). After this, uncertainties flagged by single coders were discussed in the group until agreement. *Likelihood of sepsis-related death:* The manual for evaluation of sepsis-related death is presented in Table 1, a 5-point scale ranging from *very likely* to *not sepsis-related*, drafted in consensus among the authors, after a pilot study. *Very likely* and *likely,* grouped as *sepsis-related,* imply infection as the immediate cause of death, respectively without or with contributing causes to death, such as a pre-existing end-stage condition or a concurrent critical illness such as a stroke. *Credible* and *not excludable,* grouped as *possibly sepsis-related,* imply that the infection contributed or may have contributed to death but was not obviously the immediate cause of death. *Frailty*: We applied the Rockwood Clinical Frailty Scale (CFS), Version 2.0 (EN). The CFS defines levels of frailty based on dependence in activities of daily living (ADLs). All individuals in our study were considered independent in ADLs if not explicitly described otherwise. For individuals receiving home nursing or residing in a nursing home, the hospital’s electronic health record (EHR) includes access to a scoring system for ability and dependence in ADLs, filed from their caregiver at hospital admission. This enabled retrospective, premorbid CFS evaluation. For the remaining individuals, descriptions of premorbid functional status were usually available through recent hospital follow-ups for chronic conditions, such as cancer, or in index visit chart notes to corroborate clinical decision-making, e.g., limitations of care. Although many had advanced medical conditions with limited expected lifetime, a score of 9 was rarely used, solely where they were explicitly described as very near the end of life regardless of the current infection. See Appendix 1 for further notes on frailty assessment. *Comorbidity*: Assessed with the original Charlson Comorbidity Index (CCI), weighted but not age-adjusted unless specified. Supplemented with other essential medical conditions not included in the CCI (e.g., neuromuscular disease), and definitions for immunosuppression and end-stage conditions applied by Rhee et al*.* [8], the latter adapted from Hospice Eligibility Criteria from the Centers for Medicare & Medicaid Services. The number of daily medications was collected from the patient’s admission summary. *SOFA score:* The worst recorded values 24 h ± of suspected infection, defined as the time blood cultures were drawn and/or antibiotics administered. All missing data were assumed to be normal. In most cases with respiratory failure, arterial blood gases were available, but if not, SpO2 values were used [17]. If more than one infection was diagnosed and treated during the same hospital stay, the last infection was assessed. The quick SOFA (qSOFA) score at admission was collected if the infection was present at admission. *Characteristics of infections*: If several sites of infection were evaluated and investigated, but without obvious conclusion, “unknown” was applied, also if ICD-10 code indicated otherwise. Infection related to procedures did not include minor procedures such as urinary catheterization or peripheral venous catheterization. *Patient declined further treatment:* If the patient declined, e.g., intubation, noninvasive ventilation, or even antibiotic treatment, when recommended, and at the time was deemed to have the capacity to consent. *Cluster cut-offs:* The clusters applied in *Main results* were defined by consensus among the authors, attempting to categorically describe clinically relevant conditions, to clearly illustrate the study and control populations. We used the established cut-offs for severe frailty and comorbidity according to the CFS (≥ 7) and CCI (≥ 5), as well as cut-offs in line with the previous comparable publications, such as the end-stage disease definition [8] and a CFS score of 6 [9].

**Table 1:** Evaluation manual for likeliness of sepsis-related death

Sepsis-related death	Very likely	Infection as immediate cause of death, and	No other contributory cause of death	Cholecystitis with multiple organ dysfunction, hypotensive shock
	Likely	Infection as immediate cause of death, and or do not recover from organ failure caused by infection, and	Other contributory cause(s) of death	Neutropenic bacteremia and progressive multiple organ dysfunction during treatment for aggressive multiple myeloma
Possibly sepsis-related death	Credible	Infection at time of death or during hospital admission, but of uncertain contribution to death, and	Other contributory cause(s) of death	Hospital acquired pneumonia during admission for advanced heart failure with multiple organ dysfunction
	Not excludable	Infection could be contributing to death, but no certain diagnostic findings, e.g., radiology or microbiology, and	Other contributory cause(s) of death	Treated for clinically suspected pneumonia, radiology shows lung metastases, proceeding with palliative care
Excluded from analysis	Not sepsis-related	No infection at time of death, and	Other certain cause(s) of death	Intracerebral hemorrhage, treated for hospital acquired pneumonia, 3 weeks later during same admission new, fatal intracerebral hemorrhage

Evaluated for all patients treated for infection during a hospital admission resulting in death. Illustrative case summary to the right

### Ethics approval

The study was evaluated by the Regional Committee for Medical and Health Research Ethics in Northern Norway (case number 102804) before being approved by the hospital trust data protection officer (case number 149).

### Analysis

Data were coded in Microsoft Excel for Mac version 16.52. Simple randomizations for the control populations were executed in Excel. Data were analyzed in Stata, version 17.0, MP-parallel edition. Continuous variables with non-normal distributions were compared with the Mann–Whitney *U* test. Venn diagrams were drawn using DisplayR, the online version available May 2022 and January 2023.

## Results

### Study population

We found 315 sepsis-related or possibly sepsis-related deaths out of 633 hospital deaths (50%) in our region. Of these 315 deaths, 179 (28% of all hospital deaths) were evaluated as sepsis-related, of which 73 (41%) were evaluated as very likely sepsis-related, and 106 (59%) were evaluated as likely sepsis-related. The remaining 136 cases (21% of all hospital deaths) were evaluated as possibly sepsis-related; 89 (65%) as credibly sepsis-related, whereas the last 47 (35%) deaths could not be excluded as sepsis-related. The number of cases identified from discharge ICD-10 codes was 300; in addition, 18 were identified through brief chart reviews. Out of 59 adults transferred from the regional hospital’s ICU to a university hospital ICU, two patients were transferred due to infection (i.e., in need of extracorporeal membranous oxygenation, ECMO) and died at the university hospital; these were included. Five of these 320 deaths were evaluated as undoubtedly not sepsis-related after chart reviews, and excluded from further analysis, leaving the 315.

### Characteristics of study subjects

The baseline characteristics of the study population and its subgroups (*clusters*) are presented in Table 2. The median age in the study population was 80 years. Altogether, one-third (35%) were 85 years or older; 16% were younger than 70 years. The median CFS score was 6, 91% were living with frailty (CFS ≥ 4), and 33% were living with severe frailty (CFS ≥ 7). One in four (24%) were admitted from nursing homes. Hospital stays the year prior to the admission resulting in death were common: 71% had one or more (median 3) hospital stays, with a median of 16 days in hospital that prior year. When categorizing comorbidity with the CCI, 40% were living with no to mild comorbidity (CCI 0–2), 28% with moderate comorbidity (CCI 3–4), and 32% with severe comorbidity (CCI ≥ 5). Furthermore, 30% were living with an end-stage condition prior to their last admission, 28% had cancer, and 17% were immunosuppressed. Male sex was overrepresented both overall (57%) and in each subgroup (57–64%). The male patients were slightly younger (mean age 77.5 vs. 80, *p* = 0.05), with lower CFS scores (mean 5.3 vs. 5.7, *p* = 0.04) than the female patients, there was no significant difference in CCI between the sexes.

**Table 2:** Baseline characteristics

	All	Cluster 1	Cluster 2	Cluster 3
	*N* = 315 (100%)	*N* = 229 (73%)	*N* = 47 (15%)	*N* = 39 (12%)
Age	80 (15 |27–98|)	83 (15 |43–97|)	80 (10 |54–84|)	73 (23 |33–79|)
Age ≥ 85	108 (34%)	108 (47%)	–	–
Age < 70	51 (16%)	31 (14%)	7 (15%)	13 (33%)
Female sex	136 (43%)	99 (43%)	23 (49%)	14 (36%)
Clinical frailty scale, CFS	6 (3)	6 (2)	6 (2)	4 (1)
0–3	27 (9%)	9 (4%)	6 (13%)	12 (31%)
4	65 (21%)	41 (18%)	7 (15%)	18 (46%)
5	47 (15%)	32 (14%)	6 (13%)	9 (23%)
6	71 (23%)	43 (19%)	28 (60%)	–
7–9	104 (33%)	104 (45%)	–	–
Living condition				
Home without home nursing	120 (38%)	67 (29%)	18 (38%)	35 (90%)
Home with home nursing	118 (37%)	91 (40%)	22 (49%)	4 (10%)
Nursing home	77 (24%)	71 (31%)	6 (13%)	0
Hospitalized prior year	223 (71%)	172 (75%)	29 (62%)	22 (56%)
Number of stays	3 (5)	3 (5)	4 (6)	2.5 (3)
Days hospitalized	16 (22)	15 (23)	17 (24)	16 (20)
Charlson comorbidity index, CCI	3 (4)	3 (4)	3 (6)	2 (4)
0–2, mild comorbidity	126 (40%)	83 (36%)	15 (32%)	28 (72%)
3–4, moderate comorbidity	88 (28%)	64 (28%)	13 (28%)	11 (28%)
≥ 5, severe comorbidity	101 (32%)	82 (36%)	19 (40%)	–
CCI, age adjusted	7 (4)	7 (3)	7 (6)	5 (4)
COPD	103 (33%)	67 (29%)	18 (38%)	18 (46%)
Diabetes	93 (30%)	62 (27%)	23 (49%)	8 (21%)
Heart failure	90 (29%)	68 (30%)	15 (32%)	7 (18%)
Prior stroke/TIA	78 (25%)	59 (26%)	15 (32%)	4 (10%)
Prior myocardial infarction	77 (24%)	54 (24%)	14 (30%)	9 (23%)
Dementia	53 (17%)	48 (21%)	4 (9%)	1 (3%)
Peripheral vascular disease	44 (14%)	29 (13%)	13 (28%)	2 (5%)
Rheumatological disease	36 (11%)	22 (10%)	10 (21%)	4 (10%)
Moderate–severe kidney disease*	20 (6%)	9 (4%)	10 (21%)	1 (3%)
Cancer	87 (28%)	78 (34%)	5 (11%)	4 (10%)
Other advanced condition, e.g., neuromuscular disease	32 (10%)	21 (9%)	6 (13%)	5 (13%)
End-stage condition	93 (30%)	93 (41%)	–	–
Immunosuppression	54 (17%)	43 (19%)	7 (15%)	4 (10%)
Daily medications	7 (6)	7 (4)	9 (6)	5 (10)
Polypharmacy, ≥ 5	234 (75%)	172 (75%)	39 (83%)	23 (59%)
Major polypharmacy, ≥ 10	81 (26%)	53 (23%)	22 (47%)	6 (15%)

Values are number (proportion) or median (IQR, |range|). Cluster 1: Age ≥ 85, CFS ≥ 7 or end-stage condition. Cluster 2: Age ≥ 80, CFS 6 or CCI ≥ 5. Cluster 3: Neither cluster 1 nor cluster 2

*COPD* chronic obstructive pulmonary disease, *TIA* transient ischemic attack

*Creatinine > 3 mg/dL (0.27 mmol/L), or post-kidney transplant, on dialysis or uremia

Compared to the study population, the control population consisting of sepsis survivors were significantly younger (mean age 71 vs. 79, *p* = 0.00) and had lower significantly CCI (mean 1.7 vs. 3.7, *p* = 0.00) and CFS scores (mean 4.4 vs. 5.5, *p* = 0.00). None were living with end-stage conditions. Still, 19 of 50 patients (38%) were deceased at the time of chart reviews (April 2022, 2.5–4.5 years after their septic episode). In the control population consisting of deceased patients without infection, there was no significant difference in age or CCI, CFS scores were higher than in the study population (mean 5.5 vs. 6.2, *p* = 0.02). End-stage conditions were present in 20 of 50, 40%. See table S1 for supporting information.

### Main results

**Fig. 2:**
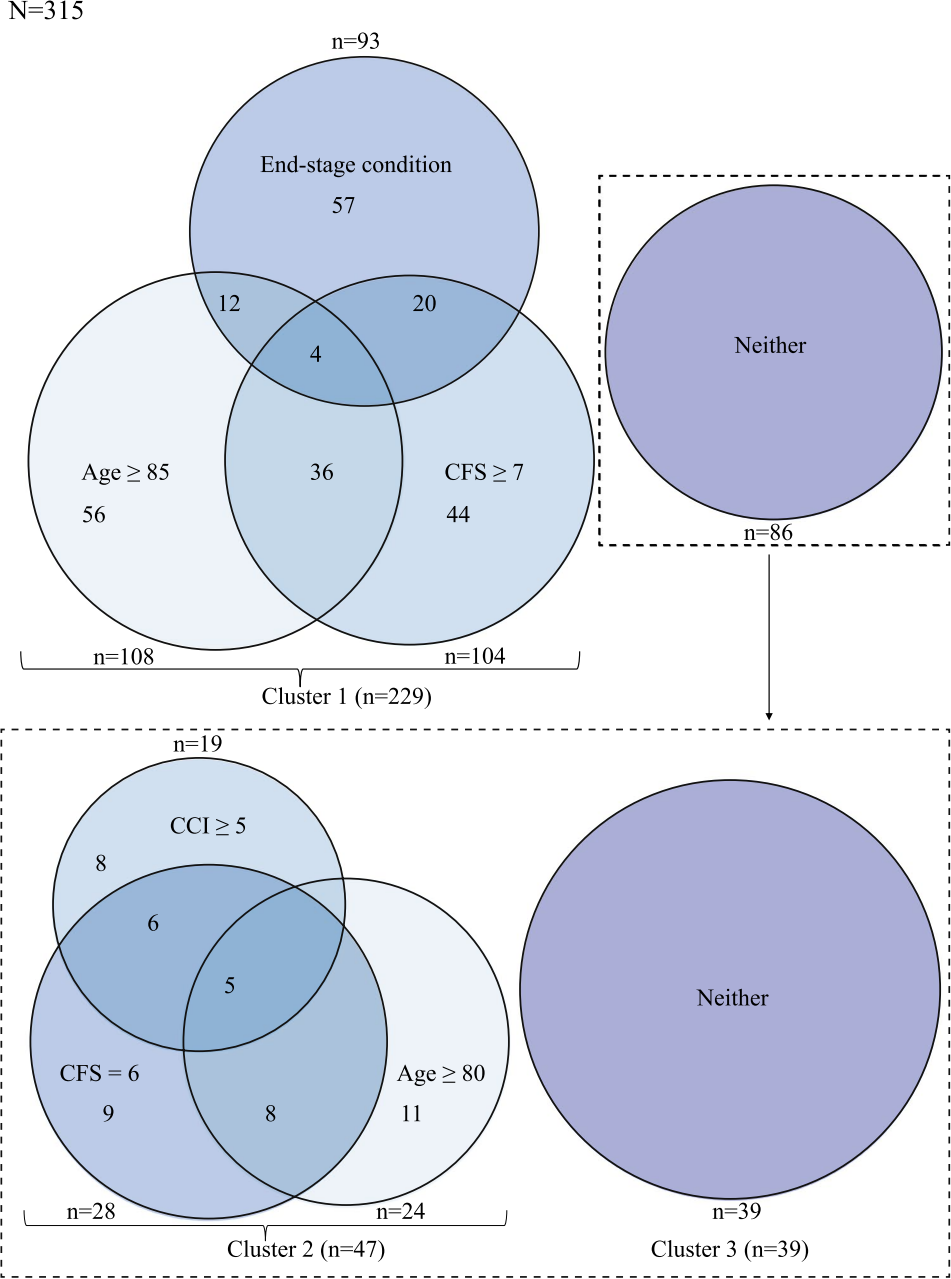
Patient characteristics in all possibly sepsis-related deaths *N* = 315

To further describe the study population, we subdivided it into three subgroups, named cluster 1–3. Cut-offs for subgrouping are discussed in *Measurements*. In cluster 1, patients fulfilled at least one of the following criteria: age ≥ 85 years, living with severe frailty (CFS ≥ 7), or living with an end-stage condition prior to hospital admission resulting in death. As presented in Fig. 2, 229 of 315 patients (73%) fell into cluster 1. Of the remaining 86 patients, 47 (15%) were grouped into cluster 2, fulfilling at least one of three more moderate but still considerable traits for burdens of age, frailty, and comorbidity: age ≥ 80 years, CFS score of 6, or severe comorbidity according to the CCI (≥ 5). The last 39 (12%) were classified into cluster 3.

As presented in Table 2, cluster 3 represents the youngest, apparently healthiest subgroup in the study population. Even so, frailty and comorbidity were still common. Only 3 of the 39 patients were younger than 70 years, living with not more than mild comorbidity (CCI < 3) and without frailty (CFS < 4). See figure S1. Further, 23 (59%) used five or more medications daily, and nearly half, 18 (46%), were living with chronic obstructive pulmonary disease (COPD).

Regarding types of admissions and infections, findings were stable among the three clusters. Regarding level of care, there was an illustrating successive increase from cluster 1 to 3, presented in Table 3 (for control populations, see table S2).

**Table 3:** Admission resulting in hospital death

	All	Cluster 1	Cluster 2	Cluster 3
	*N* = 315 (100%)	*N* = 229 (73%)	*N* = 47 (15%)	*N* = 39 (12%)
Emergency admission	292 (93%)	214 (93%)	43 (91%)	35 (90%)
Planned admission	26 (7%)	15 (7%)	4 (9%)	4 (10%)
Medical	236 (75%)	172 (75%)	35 (75%)	29 (74%)
Surgical	59 (19%)	42 (18%)	8 (17%)	9 (23%)
Neurology	12 (4%)	10 (4%)	2 (4%)	0
Orthopedic surgery	7 (2%)	5 (2%)	2 (4%)	0
Gynecology	0	0	0	0
Ear–nose–throat	1 (0%)	0	0	1 (3%)
Infection present at admission	219 (70%)	157 (69%)	35 (74%)	27 (69%)
Infection acquired during admission	96 (30%)	72 (31%)	12 (26%)	12 (31%)
Site of infection				
Airways	172 (55%)	127 (55%)	24 (51%)	21 (54%)
*Aspiration pneumonia*	*29 (9%)*	*20 (9%)*	*5 (11%)*	*4 (10%)*
Skin or soft tissue	7 (2%)	5 (2%)	2 (4%)	0
Urinary tract	18 (6%)	16 (7%)	1 (2%)	1 (3%)
Abdominal	25 (8%)	15 (7%)	6 (13%)	4 (10%)
Unknown	83 (26%)	62 (27%)	11 (23%)	10 (26%)
Foreign body, e.g., pacemaker	1 (0%)	1 (0%)	0	0
Central nervous system	2 (1%)	0	1 (2%)	1 (3%)
Other, e.g., endocarditis	7 (2%)	3 (1%)	2 (4%)	2 (5%)
Infection following a procedure				
Most likely	17 (5%)	9 (4%)	3 (6%)	5 (13%)
Possibly	22 (7%)	15 (7%)	6 (13%)	1 (3%)
Highest level of care				
Ward	102 (32%)	93 (40%)	9 (19%)	0
Intermediate ICU	145 (46%)	112 (49%)	21 (45%)	12 (31%)
ICU	67 (21%)	23 (10%)	17 (36%)	27 (70%)
ED	1 (0%)	1 (0%)	0	0
LOS	6 (10)	6 (8)	7 (11)	11 (16)
LOS intermediate ICU, if any	2 (3)	2 (3)	4 (7)	4 (11.5)
LOS ICU, if any	7 (16)	4 (6)	6 (11)	14 (16)
DNA-CPR prior to admission	58 (18%)	54 (24%)	4 (9%)	0
DNA-CPR during admission	240 (76%)	170 (74%)	36 (76%)	32 (82%)
Patient declined further treatment	20 (6%)	13 (6%)	3 (7%)	4 (10%)
Location at death				
Ward	179 (57%)	152 (66%)	22 (47%)	5 (13%)
Intermediate ICU	84 (27%)	61 (27%)	13 (28%)	10 (26%)
ICU	49 (16%)	14 (6%)	11 (23%)	24 (62%)
ED	1 (0%)	1 (0%)	0	0
Radiology	2 (1%)	1 (0%)	1 (2%)	0
QSOFA in ED ≥ 2, if infection at admission	83 (38%)	59 (38%)	18 (51%)	7 (26%)
Rise in SOFA score ≥ 2, ± 24 h. From suspicion of infection	219 (70%)	150 (66%)	37 (79%)	32 (82%)
If community acquired infection	161 (74%)	109 (69%)	28 (80%)	24 (89%)
If hospital acquired infection	58 (60%)	41 (57%)	9 (75%)	8 (66%)
If likely sepsis-related death	149 (82%)	100 (80%)	25 (86%)	23 (92%)
Sepsis-related death *(very likely and likely)*	179 (57%)	125 (55%)	29 (62%)	25 (64%)
Possibly sepsis-related death *(credible and not excludable)*	136 (43%)	104 (45%)	18 (38%)	14 (36%)

Values are number (proportion) or median (IQR). Cluster 1: Age ≥ 85, CFS ≥ 7 or end-stage condition. Cluster 2: Age ≥ 80, CFS 6 or CCI ≥ 5. Cluster 3: Neither cluster 1 nor cluster 2

*SOFA* sequential organ failure assessment, *qSOFA* quick SOFA, *ED* emergency department, *LOS* length of stay, *ICU* intensive-care unit, *DNA-CPR* do not attempt cardiopulmonary resuscitation, *ECMO* extracorporeal membrane oxygenation

**Fig. 3:**
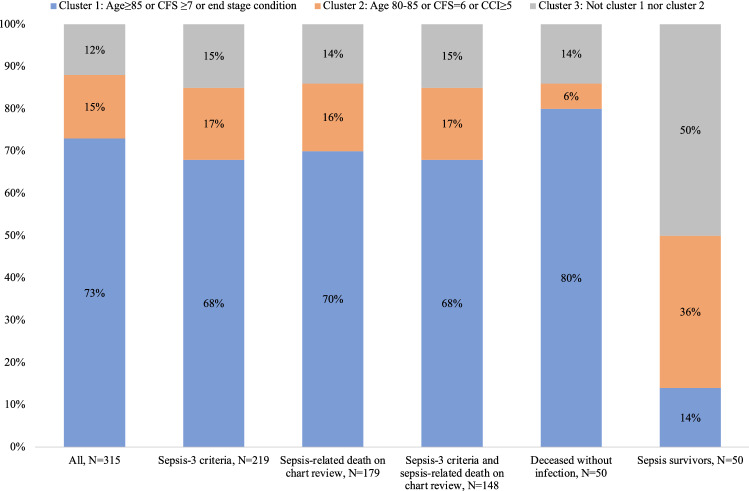
Proportion of patients in cluster 1, 2 and 3 given different sepsis definitions. Sepsis-3 criteria: Rise in SOFA score by 2 points or more 24 h ± suspicion of sepsis

If limiting the population to sepsis-related deaths on clinicians’ evaluation (179 of 315), to those fulfilling sepsis-3 criteria ± 24 h from suspicion of infection (219 of 315), or to those with sepsis-related death and fulfilling sepsis-3 criteria ± 24 h from suspicion of infection (148 of 315), the proportions of patients who classified into each cluster remained stable. This is presented in Fig. 3, alongside the proportions of patients in each cluster for both control populations.

Table 4 presents age, frailty, comorbidity, and case summary for the 25 patients in cluster 3 whose deaths were evaluated as sepsis-related. Nine of 25 died without limitations of care due to premorbid conditions. The remaining were deemed ineligible for escalation of treatment due to premorbid conditions. Of the nine patients without limitations of care, one was put on ECMO, five had severe comatose conditions, i.e., hypoxic brain injury following CPR or central nervous system (CNS) infections, one was too unstable for necessary surgical intervention, one was not eligible for additional surgery after a longer trajectory of surgical complications, and one did not want intubation. Of the remaining 16, 4 were intubated during respiratory failure but remained unstable and were not eligible for further escalation of treatment, and the last 12 were not eligible for a first or second intubation and/or necessary surgical intervention due to premorbid frailty and/or comorbidity.

**Table 4:** Age, frailty, comorbidity, and case summary for patients in cluster 3 with sepsis-related death

Age	CFS	Comorbidity	Summary
70–79	3	AF, CAD, CKD3, COPD, HF, diabetes	Cholecystitis, ERCP with duodenal perforation stented, pancreatitis and circulatory collapse first post operative day, aspiration and heart arrest during intubation, resuscitated but remains unstable, ineligible for ECMO
60–69	3	Alcohol dependence, AF	Pneumonia with respiratory failure, radiology suggests undiagnosed advanced pulmonary fibrosis and emphysema, intubated, extubated, progressive respiratory failure, ineligible for new intubation
70–79	3	CKD3, HF, arthritis	Respiratory failure in laryngal edema caused by neck abscess, heart arrest during difficult intubation, hypoxic brain injury after long CPR-time
70–79	3	AF, COPD, prior pulmectomy due to lung cancer (cured)	Pneumonia following recent pneumonia requiring intubation, patient do not want new intubation
70–79	3	COPD, localized prostate cancer, polyneuropathy	Multiple rib fractures and pneumonia, septic shock at admission, resuscitated, progressive multiple organ dysfuntcion, ineligible for intubation
60–69	3	Multiple sclerosis (mild), cachexia	UTI complicated with abscesses, several operations with complications as perforated ureter and pneumothorax, intubated, extubated, progressive multiple organ failure, ultimately ineligible for additional surgical interventions or new intubation
60–69	3	Diabetes nephropathty and retinopathy, prior MI	Septic shock at admission, after two weeks being sick at home with viral infection and secondary pneumonia, ARDS, ECMO > 10 days
60–69	4	CTD, HT, multiple myeloma first relapse after stem cell treatement	Bacteremia during chemotherapy, concurrent bleedings and hyperviscosity, aggressive cancer development, progressive multiple organ dysfunction, ineligible for intubation
50–59	4	AF, HF, prior MI, recent ischemic stroke	Ischemic stroke after recent ischemic stroke, progressive heart failure complicated with pneumonia and lung abscesses not responding to therapy, ineligible for intubation
50–59	4	AF, aortic graft, COPD, HF, Marfan syndrome, PM, prostehetic heart valve	Bilateral pneumonia, hypoxic respiratory arrest at the ward, rescusitated and intubated, but do not wake up, hypoxic brain injury after long CPR-time
70–79	4	Leukemia	Septic shock and ARDS during during chemotherapy, heart arrest, rescusitated and intubated but remains unstable, withdrawn treatment due to very poor prognosis
70–79	4	Aortic stenosis, peripheral vascular disease, prior MI	Bacterial meningitis, comatose, intubated, but severe brain damage, do not wake up
70–79	4	CAD, aortic graft, localized cancer urinary bladder and rectum	Disseminated mycobacterial infection following cancer treatment, multiple organ dysfunction, intubated, other organs improves but remains comatose
70–79	4	COPD with chronic respiratory failure	Pneumonia with respiratory failure, kidney failure, intubated, complicated with intestinal ischemia, ineligible for surgery
70–79	4	Asthma, HT	Bacteremia and candidemia following multiple surgery for abdominal abscesses after diverticulitis with complications as anastomotic leaks and ileus, ultimately ineligible for addtional surgery
70–79	4	AAA, AF, HT, mitral insufficiency	Operated AAA, 5th day post operative rapid onset multiple organ faliure, intubated, heart arrest, unsuccessful resuscitation, long CPR-time
70–79	4	CAD, COPD with chronic respiratory failure, diabetes, obesity-hypoventilation syndrome	Viral infection complicated with bacterial pneumonia, progressive respiratory failure, ineligible for intubation
60–69	4	Alcohol dependence, asthma, cachexia, epilepsy	Pneumonia with respiratory failure, ineligible for intubation
70–79	4	Arthritis, CAD, peripheral vascular disease, polyneuropathty, prior TIA	Pneumonia with respiratory failure, intubated, ARDS, ineligible for ECMO
70–79	4	COPD, HT, prior TIA	Viral infection complicated with bacterial pneumonia, intubated but progressive respiratory failure, ineligible for ECMO
70–79	5	COPD, cachexia, epilepsy, prior ischemic stroke	Dysphagia and rapid weight loss, suspected neuromuscular disease, recurrent pneumonias most likely due to aspirations, ineligible for intubation
70–79	5	CAD, HT, lymphoma, recent bilateral PE, rheumatoid arthritis	Chylothorax and pneumonia during chemotherapy, concurrent UTI and spontaneous muscular bleedings, progressive respiratory failure, ineligible for intubation
60–69	5	Alcohol dependence, COPD with chronic respiratory failure, HT	Pneumonia, prehospital respiratory arrest, short CPR-time, intubated, extubated before ileus and progressive respiratory failure, ineligible for surgery and new intubation
70–79	5	Alcohol dependence, COPD with chronic respiratory failure, ongoing significant weight loss	Pneumonia with respiratory failure and heart failure, suspected malignancy but too unstable for investigations, ineligible for intubation
70–79	5	CKD4, colostomy, PM, prior year general malaise but have not wanted help	Bacteremia in cholangitis, severly distended gall bladder, “moribund at admission” in septic shock, intubated but too unstable for surgical intervention

*AAA* abdominal aortic aneurysm, *AF* atrial fibrillation, *CAD* coronary artery disease, *CKD* 1–5 chronic kidney disease stage 1–5, *COPD* chronic obstructive pulmonary disease, *CTD* connective tissue disease, *ECMO* extracorporeal membrane oxygenation*,* *ERCP* endoscopic retrograde cholangiopancreatography, *HT* hypertension, *HF* heart failure, *MI* myocardial infarction, *PM* pacemaker, *TIA* transient ischemic attack, *UTI* urinary tract infection

Assessing current sepsis scoring tools, 38% of the 219 who presented to the ED with an infection that they died from or with had a positive qSOFA score of 2 or more, and 74% had a rise in SOFA score by 2 points or more during the first 24 h. If the infection was acquired during the admission, 60% had a rise in SOFA score by 2 points or more in the 24 h before and after the suspicion of infection.

## Discussion

We have examined all possibly sepsis-related hospital deaths in our region for 2 years. Our main finding is the dominance of advanced age, frailty, and comorbidity in all hospital fatalities where infections contributed to death, with or without sepsis. Although younger and generally healthy adults also die from sepsis, fortunately, these seem to be very rare exceptions in our population.

In our study, 88% were living with considerable frailty, advanced age, and/or had severe comorbidities. This corresponds to previous findings in other populations [8, 9]. However, even among the remaining 12%, most were living with conditions that on chart reviews seemed to be important contributors to death, as presented in Table 4. While not everyone had severe comorbidities, several factors limited their functional reserves. These factors were not always easy to categorize, yet they were clear to clinicians studying the patient’s charts, corroborated by the finding that most died with documented limitations of care. Our findings suggest that while sepsis is a serious condition that requires prompt and correct treatment in all patients, death mainly occurs among those already at significantly increased risk. This is illustrated by the similarities between the study population and the patients deceased from non-infectious diseases (Fig. 3).

We chose a broad inclusion strategy with a generous definition of possibly sepsis-related deaths to avoid missing any cases. We did not solely use the Sepsis-3 criteria to define sepsis. As all patients died in our study population, we would argue that all have possible sepsis as they have both an infection and organ failure. As with other definitions of sepsis, the challenge is to determine the extent to which infection is the cause of death, particularly in a population with severe comorbidities and frailty. Narrowing down the population to only those whose deaths were evaluated as likely or very likely sepsis-related, our finding remained consistent. This was also the case when including only those fulfilling the Sepsis-3 criteria. Thus, our findings hold true both for a broad and a narrower definition of sepsis.

Our study has limitations. It was retrospective and not blinded. Because of this, we strived to be compliant with guidelines for retrospective chart reviews [18], performing a pilot study, drafting robust case definitions, training abstractors, and assessing raters’ agreement. We assumed all missing data, from vital signs and blood tests to descriptions of abilities in ADL, to be normal to avoid overestimating the burdens of comorbidity and frailty in our population. We included all possibly sepsis-related deaths in the analysis, partly because of the lack of abstractor blinding. It is a single-center study, and its generalizability depends on the extent to which our hospital population reflects those in other regions. However, ours are the only hospitals in the region, and all emergency admissions will be to these hospitals, so it is difficult to see a systematic loss of septic patients. One exception is patients discharged to end-of-life care at a nursing facility. We did not access accurate data regarding the proportions of such discharges. But through chart reviews to identify sepsis survivors, we found that 4 of 342 patients discharged alive after a hospital stay with an infectious disease, fulfilled sepsis-3 criteria during the admission, and had died within 30 days. Two of these were discharged to palliative care, suggesting that such discharges do not constitute a large proportion of discharges following sepsis. Nevertheless, the lack of these cases in our study population would typically contribute to underestimate the burden of premorbid conditions, rather than the opposite. Further, both control populations are relatively small regarding statistical differences between the groups. They were included primarily to investigate if there was in fact a different patient population served by our hospitals other than our study population, i.e., the sepsis survivors, and to contextualize.

Our data are not applicable to low- and middle-income countries, and some factors might affect the comparability to other high-income countries. First, Norway has an easily accessible, universal public health care system, and high standards of living. Second, all patients, unless severely ill, will first be screened by a general practitioner out of hospital, causing less crowding and relatively short waiting times in the emergency departments (EDs). In the study period, 70–90% of patients triaged as potentially septic received antibiotics within 1 h after arrival at the EDs or before arrival (numbers from local quality registers). Finally, Norway has low antimicrobial resistance rates [19], so most infections are adequately covered with narrow-spectrum empiric therapy.

Still, our population seems to correspond with other studies from high-income countries, also regarding estimated proportions of sepsis-related deaths. We evaluated 28% of all hospital deaths to be sepsis-related (i.e., infection as the immediate cause of death), and up to 50% were possibly sepsis-related. Liu et al*.* reported that sepsis was present in 35–56% of hospital deaths through ICD coding [3], while Rhee et al*.* found sepsis in 50% of hospital deaths through chart reviews [8], with sepsis as a direct cause of death in 35%. Both studies are from the US. Our estimates are, however, not in line with the previous data from Norway. Knoop et al*.* found sepsis-related deaths to constitute 13% of all hospital deaths during 2011–2012 [5], but in a solely ICD code-based study, which may contribute to the discrepancy [20, 21].

Finally, our data could be explained by an altogether very old, frail, and comorbid population served by our hospitals. The sepsis survivor population indicates that there is in fact a less burdened hospital population than the study population. However, the sepsis survivors are not a particularly young, healthy group either: 19 of 50 were deceased within 2.5–4.5 years. Yet, our study population does not seem to have greater burdens of premorbid conditions than the previously referred to studies: Kopczynska et al*.* found CFS scores of six or more in 78% of patients in ward populations whose deaths were attributable to sepsis [9]. In our study, this portion was 56% overall, 58% among those with sepsis-related deaths, and 71% among those whose deaths were sepsis-related and who died on the wards. Rhee et al*.* found end-stage conditions present at the time of admission among 40% of those whose deaths were sepsis-related in their US hospital population [8], versus 30% in our study population.

## Conclusions

Our findings indicate that in our and similar populations, strikingly few sepsis-related deaths occur among adults living without considerable burdens of comorbidity, frailty, and advanced age. In our data, sepsis could be seen more as the back-breaking straw in a vulnerable individual, than a force taking one from good health to death. If this holds true for other high-income settings, it seems relevant to question the extent to which sepsis survival in such populations can be significantly improved. By extension, could other informative endpoints besides mortality, such as residual organ dysfunction and functional decline, gain ground in future sepsis studies? Ultimately, our findings can also elucidate the value of preventing infections from occurring in vulnerable individuals, such as vaccinations and prevention of healthcare-associated infections.

## Supplementary Information

Below is the link to the electronic supplementary material.

Supplementary file1 (DOCX 17 KB)

Supplementary file2 Venn Diagram, Patient Characteristics, Cluster 3 (EPS 740 KB)

Supplementary file3 (DOCX 28 KB)

## Data Availability

The datasets used and analyzed during the current study are available from the corresponding author on reasonable request.
