# Correction: Patient characteristics in sepsis-related deaths: prevalence of advanced frailty, comorbidity, and age in a Norwegian hospital trust

**DOI:** 10.1007/s15010-023-02046-3

**Published:** 2023-05-12

**Authors:** Marianne Ask Torvik, Stig Haugset Nymo, Ståle Haugset Nymo, Lars Petter Bjørnsen, Hanne Winge Kvarenes, Eirik Hugaas Ofstad

**Affiliations:** 1 Department of Infectious Disease, Nordland Hospital Trust, Parkveien 95, 8005 Bodø, Norway; 2 Department of Emergency Medicine, Nordland Hospital Trust, Parkveien 95, 8005 Bodø, Norway; 3 Department of Cardiology, Nordland Hospital Trust, Parkveien 95, 8005 Bodø, Norway; 4 Emergency Department, Clinic for Emergency Medicine and Prehospital Care, St. Olav’s Hospital University Hospital, NTNU, ISB, Prinsesse Kristinas Gate 3, 7030 Trondheim, Norway; 5 Faculty of Health Sciences, UiT the Arctic University of Norway, Hansine Hanses Vei 14, 9037 Tromsø, Norway

**Correction: Infection** 10.1007/s15010-023-02013-y

In this article the affiliation ‘UiT the Arctic University of Norway, Faculty of Health Sciences, Hansine Hanses vei 14, 9037 Tromsø. Norway’ for authors Marianne Ask Torvik, Stig Haugset Nymo, Ståle Haugset Nymo and Eirik Hugaas Ofstad were missing.

The original article has been corrected.

