# Supplementary material for: Patient characteristics in sepsis-related deaths: prevalence of advanced frailty, comorbidity, and age in a Norwegian hospital trust
Source: Infection. 2023 Mar 9;51(4):1103–15. doi: 10.1007/s15010-023-02013-y (PMC10352435; doi:10.1007/s15010-023-02013-y)
Supplement: Supplementary file 1 — Supplementary file1 (DOCX 17 KB) [file 15010_2023_2013_MOESM1_ESM.docx]

**APPENDIX 1: DERIVING CLINICAL FRAILTY SCALE SCORES FROM DESCRIPTIONS OF ABILITIES IN ACTIVITIES OF DAILY LIVING**

We applied the Rockwood Clinical Frailty Scale (CFS), Version 2.0 (EN). The CFS defines levels of frailty based on dependence in activities of daily living (ADLs). All individuals in our study were considered independent in all ADLs if not explicitly described otherwise.

For individuals who did not receive home nursing services, descriptions of premorbid functional status were usually available through recent hospital follow-ups for chronic conditions, or in index visit chart notes to corroborate clinical decision-making, e.g., limitations of care. If **no** descriptions were available, they were scored 2 or 3: 2 if they were presumably fit, e.g., a tourist without prior medical problems, or 3 if they had prior medical problems that seemed to be “well controlled”, i.e., without recent medical contacts. To score 1, they should be explicitly described as such, both corresponding to “among fittest for their age”, and without apparent slowing or need for assistance. E.g., a 97-year-old could be described as very fit for their age, but still as living with frailty, and would be scored according to abilities in ADLs. A score of 4 requires information about the burden of symptoms before the acute illness, described as being “slowed up” or “tired during the day”. If this was not explicitly available, but they applied walking aids such as a walker, or were described as e.g., “inactive” or “rarely outside”, or had frequent medical contacts during the prior year, they were scored 4 or higher.

Most individuals receiving home nursing services scored between 4 and 7. For individuals receiving home nursing services or residing in a nursing home, the hospital’s electronic health record (EHR) includes access to a scoring system for ability and dependence in ADLs, filed from their caregiver at hospital admission. This scoring system is a national register for nursing and care services, called IPLOS (*Individbasert pleie- og omsorgsstatistikk*/ *Individual-based nursing and care statistics*). The IPLOS comprises 17 variables based on the International Classification of Functioning, Disability and Health (ICF), where the person receiving services is scored from 1 to 5 for each variable. A score of 3 or more indicates an increasing need for assistance, where 3 marks the need for personal assistance and 5 describes complete dependence. A score of 9 can be registered if the variable is not relevant to the service provided. The 17 variables are housework, obtaining goods and services, personal hygiene, dressing, toilet, cooking, eating, moving indoors, moving outdoors, taking care of own health, memory, communication, decision making, finances, social participation, behavior control, sight, hearing, experienced safety, and ability to take initiative. These variables correspond well with the CFS category descriptions. If someone only received e.g., house cleaning and/ or medications delivered to their house every two weeks, they could be scored lower than 5, but if they received daily services, they would be scored 5 or higher. Specifically, we evaluated assistance with compression stockings, wound and stoma care as “medications” and not “dressing” or “bathing”, so individuals receiving these services did not necessarily score 6 or higher. To do so, they should require assistance as described in the CFS for a score of at least 6: in all outside activities, and inside with stairs, bathing, or dressing. However, in many cases, a score between 6 and 7 seemed most fitting, as they required personal assistance in a wide range of ADLs. But a score of 7 describes someone “completely dependent for personal care”, so if there was any doubt regarding someone’s complete dependence, a score of 6 was assigned.

Most individuals living in a nursing home scored 7 or higher. This was also based on the IPLOS scores and/or other available descriptions, they were not assumed completely dependent for personal care because of their living situation. A score of 8 was assigned if they were bedridden, either at home or at an nursing home.

Although many had advanced medical conditions with limited expected lifetime, a score of 9 was rarely used, solely where they were explicitly described as very near the end of life regardless of the current infection.
