# Supplementary material for: Patient characteristics in sepsis-related deaths: prevalence of advanced frailty, comorbidity, and age in a Norwegian hospital trust
Source: Infection. 2023 Mar 9;51(4):1103–15. doi: 10.1007/s15010-023-02013-y (PMC10352435; doi:10.1007/s15010-023-02013-y)
Supplement: Supplementary file 3 — Supplementary file3 (DOCX 28 KB) [file 15010_2023_2013_MOESM3_ESM.docx]

Table S1. Baseline characteristics, control populations

|  | Sepsis survivors  N=50 | Deceased without infection  N=50 |
| --- | --- | --- |
| Age Age ≥85 Age <70 | 72 (21 \|29-101\|) 11 (22%) 21 (42%) | 80.5 (13 \|39-99) 15 (30%) 7 (14%) |
| Female sex | 23 (46%) | 26 (52%) |
| CFS  0-3 4 5 6 7-9 | 5 (3) 24 (44%) 2 (4%) 13 (26%) 4 (8%) 9 (18%) | 7 (5) 7 (14%) 6 (12%) 8 (16%) 3 (6%) 26 (52%) |
| Living condition Home without home nursing Home with home nursing Nursing home | 26 (52%) 20 (40%) 4 (8%) | 19 (38%) 21 (42%) 10 (20%) |
| Hospitalized prior year Number of stays  Days hospitalized | 23 (46%) 2 (2) 14 (27) | 38 (76%) 3 (3) 13 (29) |
| CCI 0-2, mild comorbidity 3-4, moderate comorbidity ≥5, severe comorbidity CCI, age adjusted | 1 (3) 35 (70%) 12 (24%)  3 (6%) 5 (4) | 4 (5) 20 (40%) 9 (18%) 21 (42%) 7 (5) |
| COPD Diabetes Heart failure Prior stroke/TIA Prior myocardial infarction Dementia  Peripheral vascular disease Rheumatological disease Moderate-severe kidney disease* | 12 (24%) 14 (28%) 8 (16%) 7 (14%) 6 (12%) 6 (12%) 2 (4%) 6 (12%) 2 (4%) | 11 (22%) 13 (26%) 16 (32%) 13 (26%) 14 (28%) 11 (22%) 5 (10%) 2 (4%) 3 (6%) |
| Cancer | 1 (2%) | 21 (42%) |
| Other advanced condition,  e.g., neuromuscular disease | 3 (6%) | 1 (2%) |
| End stage condition | 0 | 20 (40%) |
| Immunosuppression | 8 (16%) | - |
| Daily medications Polypharmacy, ≥5  Major polypharmacy, ≥10 | 6.5 (6) 33 (66%) 10 (20%) | 7 (5) 37 (74%) 12 (24%) |

Values are number (proportion) or median (IQR, |range|). Sepsis survivors: Alive > 30 days from discharge after sepsis. Deceased without infection: Hospital deaths without infection. CFS Clinical frailty scale, CCI Charlson comorbidity index, COPD chronic obstructive pulmonary disease, TIA transient ischemic attack, * Creatinine >3 mg/dL (0.27 mmol/L), or post kidney transplant, on dialysis or uremia.

Table S2. Index hospital admission, control populations

|  | Sepsis survivors  N=50 | Deceased without infection  N=50 |
| --- | --- | --- |
| Emergency admission Planned admission | 49 (98%)  1 (2%) | 47 (94%) 3 (6%) |
| Medical  Surgical  Neurology  Orthopedic surgery  Gynecology Ear-nose-throat | 31 (62%) 13 (26%) 2 (4%) 2 (4%) 2 (4%) 0 | 31 (62%) 9 (18%) 4 (8%) 4 (8%) 2 (4/%) 0 |
| Infection present at admission Infection acquired during admission | 47 (94%) 3 (6%) | - |
| Site of infection  Airways *Aspiration pneumonia* Skin or soft tissue Urinary tract Abdominal  Unknown  Foreign body, e.g., pacemaker Central nervous system Other, e.g., endocarditis | 24 (48%) *1 (2%)* 5 (10%) 8 (16%) 9 (18%) 4 (8%) 0 0 0 | - |
| Infection following a procedure Most likely Possibly | 2 (4%) 3 (6%) | - |
| Highest level of care Ward Intermediate ICU ICU ED | 24 (48%) 19 (38%) 7 (14%) 0 | 23 (46%) 17 (34%) 10 (20%) |
| LOS  LOS intermediate ICU, if any LOS ICU, if any | 6 (8) 2 (2) 3 (12) | 4 (10) 1 (1) 0.5 (5) |
| DNA-CPR prior to admission DNA-CPR during admission | 4 (8%) 8 (16%) | 6 (12%) 38 (76%) |
| Patient declined further treatment | 0 | 1 (2%) |
| Location at death Ward Intermediate ICU ICU ED Radiology | - | 36 (72%) 8 (16%) 5 (10%) 0 1 (2%) |
| QSOFA in ED ≥2,  if infection at admission | 25 (50%) | - |
| Rise in SOFA score ≥2, ± 24 hrs. from suspicion of infection If community acquired infection If hospital acquired infection If likely sepsis-related death | - | - |
| Sepsis-related death  *(very likely and likely)* Possibly sepsis-related death *(credible and not excludable)* | - | - |

Values are number (proportion) or median (IQR). |). Sepsis survivors: Alive > 30 days from discharge after sepsis. Deceased without infection: Hospital deaths without infection SOFA sequential organ failure assessment, qSOFA quick SOFA, ED emergency department, LOS length of stay, ICU intensive care unit, DNA-CPR do not attempt cardiopulmonary resuscitation, ECMO extracorporeal membrane oxygenation.
